# Localized Bilateral Superior and Inferior Orbital Neurofibroma in the Absence of Neurofibromatosis

**DOI:** 10.1155/2021/6655134

**Published:** 2021-06-04

**Authors:** Daniela Rojas-Correa, Álvaro Bengoa-González, Enrique Mencía-Gutiérrez, Aurelio Hernández-Laín, Elena Salvador, Agustín Martín-Clavijo, Justino Jiménez-Almonacid, María-Dolores Lago-Llinás

**Affiliations:** ^1^Ophthalmology Department, 12 de Octubre Hospital, Complutense University, 28041 Madrid, Spain; ^2^Neuropathology Department, 12 de Octubre Hospital, Complutense University, 28041 Madrid, Spain; ^3^Radiology Department, 12 de Octubre Hospital, Complutense University, 28041 Madrid, Spain; ^4^Dermatology Department, Queen Elizabeth Hospital, Birmingham University, B15 2TH Birmingham, UK

## Abstract

Localized or isolated neurofibromas are peripheral nerve sheath tumors. They are rare in the orbit and occur without a systemic neurofibromatosis. There are few cases of bilateral tumors reported but none affecting both supraorbital and infraorbital nerves. We report a 45-year-old female who presented an extraconal mass in the right orbit as an incidental finding in a head computer tomography, without ocular symptoms. Magnetic resonance image showed a well-defined oval mass in the right supraorbital and infraorbital nerves, of similar characteristics, as well as smaller masses in the left supraorbital and infraorbital nerves. A progressive increase in size of the left supraorbital and infraorbital tumor motivated their surgical excision. The histological result was compatible with a neurofibroma. These uncommon orbital tumors are slow growing and affect the sensory nerves of the trigeminal nerve. Neurofibromas usually present progressive symptoms due to the orbital mass, proptosis, or visual changes although not in this case. Surgical removal is the only definitive treatment.

## 1. Introduction

Neurofibromas are classified within the tumors that affect peripheral nerves. Localized or isolated neurofibromas are peripheral nerve sheath tumors and are rare in the orbit, representing less than 1% of orbital tumors [1, 2]. Unlike the relatively more common plexiform neurofibromas, localized neurofibromas tend to be well circumscribed and are not typically associated with type 1 neurofibromatosis (NF) [1, 3]. These tumors can be solitary but more frequently are multiple [1]. Solitary neurofibromas in the adult orbit, in the absence of systemic NF, are difficult to clinically differentiate from other orbital tumors [4]. They are composed of Schwann cells, perineural cells, and fibroblasts [5]. Most of the few reported cases were unilateral [6, 7] with 5 cases of bilateral tumors reported in the literature [8]. Herein, we report a case of neurofibromas affecting both supraorbital and infraorbital nerve bilaterally, without association with familiar NF, a combination not reported before.

## 2. Case Presentation

Three years ago, a 45-year-old female presented an extraconal mass in the right orbit as an incidental finding in a head computer tomography scan and magnetic resonance image (MRI) during investigations following two syncopal episodes.

On examination, she had a best corrected visual acuity (BCVA) of 1.0 (decimal scale) in both eyes with no exophthalmos nor diplopia and only a minimal asymmetry of the palpebral fold between both eyes. Extraocular muscle movement was normal. There were no alterations in fundoscopy, and the intraocular pressure was normal.

**Figure 1:**
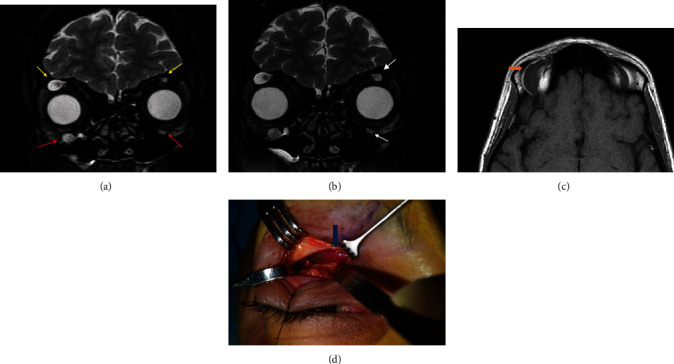
(a) Coronal MRI T2-weighted imaging showed bilateral hyperintense well-defined extraconal masses in the anterosuperior margin of both orbits (yellow arrows) (the greater one on the right side) corresponding to the supraorbital nerves. There was also another extraconal mass in the anteroinferior margin of the right orbit related to the infraorbital nerve, with similar appearance, and smaller on the left side (red arrows). (b) Growth of lesions in the left orbit (white arrows). (c) MRI axial T1-weighted imaging demonstrated the right anterosuperior tumor with isointensity relative to muscle with a fusiform morphology (orange arrow). (d) Intraoperative image of the lesion (blue arrow).

The MRI showed a well-defined hypodense oval mass in T1 and hyperintense in T2 sequencing. There was also an ill-defined area, appearing hypodense on T2 but of intermediate density in T1, measuring 24 × 9 mm (Figures 1(a) and 1(c)). The mass had very well-defined borders in the anterosuperior margin of the right orbit, probably corresponding to the supraorbital nerve. With this presentation, the initial differential diagnosis was of a vascular lesion or a schwannoma. An MRI angiogram showed no uptake of contrast, ruling out a vascular lesion, but showed a second mass in the right orbit, of similar characteristics, in the right infraorbital nerve, as well as smaller masses in the left supraorbital and infraorbital nerves (Figure 1(a)). Although the lacrimal glands and the extraocular muscles were not affected, the findings were suggestive of an IgG4-related disease; however, the serum IgG4 levels were within normal ranges.

As the masses were affecting several nerves and were asymptomatic, a watchful wait approach was adopted. During the 3 years of follow-up, MRI demonstrated the existence of similar lesions, located in the right supra- and infraorbital nerve as well as on the left side. However, there was a progressive increase in size and signal enhancement corresponding to the left supraorbital nerve during the last year, which also affected the left infraorbital nerve (Figure 1(b)), leading us to remove the tumor to make a definitive diagnosis.

**Figure 2:**
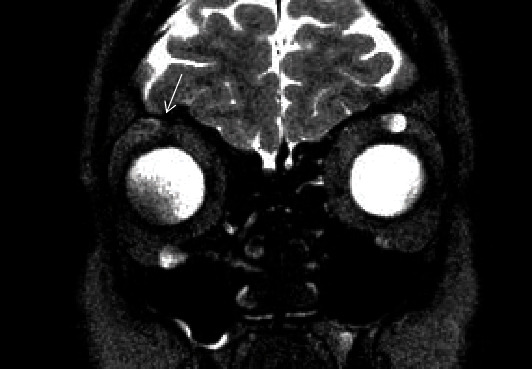
Postoperative coronal RMI T2-weighted imaging showed no recurrence in superior orbit right side (arrow). There was discrete increase in size of those located in the inferior right orbit and in the superior and inferior left orbit.

An orbitotomy with access through the upper eyelid skin crease was performed, exposing a fusiform lesion, cystic in appearance, measuring approximately 3 cm in its major axis, protruding from the supraorbital nerve and extending deeply into the orbit (Figure 1(d)). The lesion was removed en bloc. There was no deficit in the ocular mobility postsurgically nor any reduction in BCVA; the patient only presented decreased sensation and numbness in the right frontal nerve distribution area; this decreased sensation in the right forehead has not completely recovered at the last visit. MRI follow-up demonstrated no recurrence of the excised tumor and a slight increase in size of the other lesions (Figure 2).

**Figure 3:**
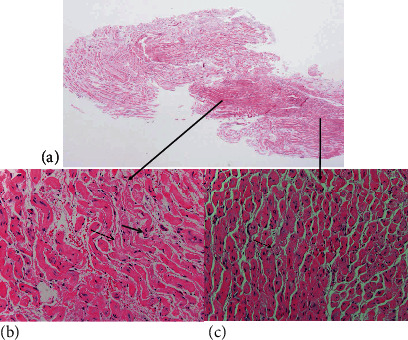
(a) Microscopic panoramic view of the lesion. Hematoxylin and eosin, 20x. (b) Heavy collagen bundles described as “shredded carrots” (thick arrow). Note the oval and wavy nuclei (thin arrow). Hematoxylin and eosin, 200x. (c) Whorls reminiscent of true onion bulbs (arrow). Hematoxylin and eosin, 200x.

**Figure 4:**
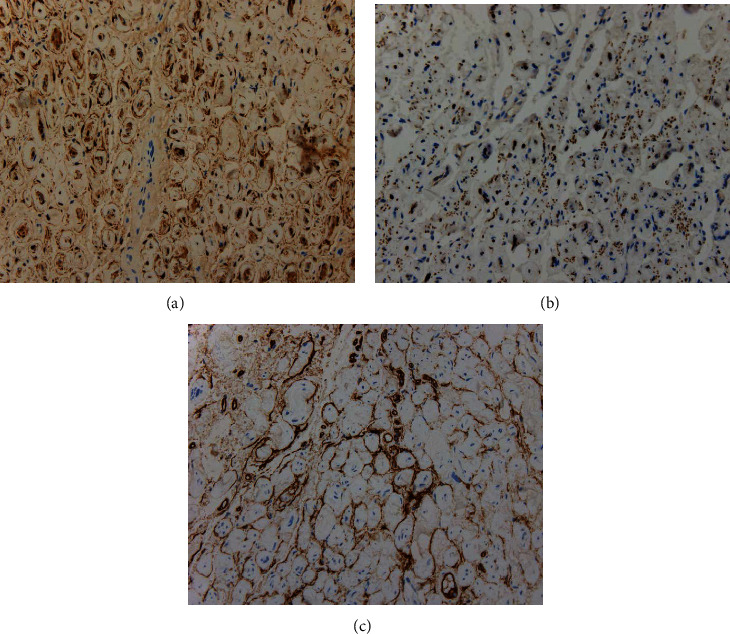
(a) S100 protein immunoreactivity showed Schwann cells. (b) Immunochemistry with neurofilaments was positive in axons. (c) Intermixed cells CD34 positive were observed.

The microscopic analysis showed a lesion with a loose connective stroma, areas with spindle and oval cells with wavy nuclei, along with dense bundles of hyalinized collagen in the form of “shredded carrots” and others composed of concentric structures of Schwann cells around axons with an “onion bulb” appearance (Figures 3(a)–3(c)). Immunohistochemistry showed positivity with S100 protein in the Schwann cells; axons inside the lesion were positive for neurofilament protein, epithelial membrane antigen (EMA), and CD34 in cells around Schwann cells. The histological findings and the presence of all the cellular elements of the nerve were compatible with a diagnosis of neurofibroma (Figures 4(a)–4(c)). No inflammatory infiltrates, plasma cells, or other data suggestive of IgG4 disease were identified.

Further investigation showed an absence of axillary/inguinal freckling, cafe au lait spots, and Lisch nodules of the iris. No first- or second-degree relatives suffered from similar illness or had any such clinical stigmata. The patient had no other dysmorphic features or endocrine syndromes and no systemic stigmata indicative of familial NF, nor any compatible family history. Due to the benign appearances of the lesion, it was decided, together with the patient, to restart the watchful wait and to intervene only if there was a significant change in size or functional deficits. None have been noted after 20 months of follow-up.

## 3. Discussion

Isolated neurofibromas of the orbit (also known as localized, solitary, or circumscribed) is the term used to refer to those neurofibromas of the orbit, which occur without a systemic diagnosis of NF1, previously known as von Recklinghausen disease [9]. Overall, neurofibromas are uncommon in adults, 1-3% of all space-occupying lesions of the orbit [2, 10]. The incidence of true isolated neurofibromas in the orbit is difficult to determine because of its relation to NF; it has been estimated that 10%-28% of all solitary neurofibromas are related to NF [6]. Some authors [6, 7, 11] described solitary neurofibromas in their series, but there is a paucity of reports of bilateral solitary neurofibromas in non-NF patients [8]. The case we present is the first to our knowledge affecting the supra- and infraorbital nerves bilaterally. As in other cases [12], our patient was middle-aged, and the tumors were slow growing, affecting the sensory nerves of the trigeminal nerve [4]. The location of tumors in the superior orbit in the distribution of the ophthalmic nerve (V1) is typical, but involvement of the maxillary nerve (V2) in the floor of the orbit, as in our case, is less frequent [1]. Garrity et al. noted multiple tumors in a single patient involving other nerves traversing the orbit [6].

Even though our patient was asymptomatic and the tumor was found as an incidental finding, patients with solitary neurofibromas usually present with a long history of progressive symptoms due to the orbital mass, a palpable mass, gradually progressing proptosis, visual changes, diplopia, hypoesthesia, or pain (although pain is less frequent) [4, 11, 12]. As a differential diagnosis, IgG4-related disease was considered [13] although the histological findings were not supportive. This illness affects primarily the lacrimal gland, but an increase in size can also be seen in other orbital structures such as the supraorbital and infraorbital nerves [14]. Suspicion of this disease arises when high serum levels of IgG4 are found. However, normal serum levels (as was our case) do not rule out the diagnosis. Other differential diagnoses included cavernous hemangioma, hemangiopericytoma, orbital varix, neurilemmoma, and fibrous histiocytoma, among others [1]. Differential diagnosis of a peripheral nerve sheath tumor encompasses schwannoma, neurofibroma, perineurioma, granular cell tumor, benign hybrid tumors, and malignant peripheral nerve sheath tumors [4, 15].

We were able to rule out familial NF. The diagnostic criteria require the presence of 2 or more of the following criteria [15]:

- Six or more cafe au lait macules over 5 mm in their greatest diameter in prepubertal individuals and over 15 mm in postpubertal individuals
- Two or more neurofibromas of any type or one plexiform neurofibroma
- Freckling in the axillary or inguinal region
- Optic glioma
- Two or more Lisch nodules (iris hamartomas)
- A distinctive osseous lesion such as sphenoid dysplasia or thinning of long bone cortex with or without pseudarthrosis
- A first-degree relative (parent, sibling, or offspring) with NF1 by the above criteria

Several imaging features, including multiplicity, multilobulation, ring-configured contrast enhancement, and MRI signal intensity heterogeneity, may point to the diagnosis of localized neurofibroma [4]. MRI can help with preoperative planning and selecting the best surgical approach should surgery become necessary [4]. Surgical resection may be the only way to arrive at a definitive diagnosis [12].

The orbital mass was biopsied via anterior orbitotomy by eyelid crease approach.

Ultrastructural studies showed that this tumor was composed of a mixture of three cell types: Schwann cells, perineural cells, and fibroblasts [7, 16]. Immunohistochemistry showed immune reactivity in EMA and S100 protein (Figures 4(a)–4(c)) in perineurioma and neurofibroma, respectively [17, 18]. Immunohistochemistry in our case was compatible with neurofibroma [15] (Table 1).

The importance of the localized neurofibroma is that the tumor can be easily removed leading to a complete surgical cure. In cases where the tumor was not completely excised, no recurrences requiring further surgery were found after long follow-up periods [7, 11]. Patients with localized neurofibromas usually see a return to normal globe position, visual acuity, and sensation after surgery. However, there is a risk for postoperative anesthesia, reported in 72% of patients with neurofibromas, likely attributable to damage to the involved sensory nerves during removal [12].

## 4. Conclusion

Neurofibromas without associated NF rarely occur in the orbit. In addition, the peculiarity of our case lies in the bilateral involvement of the supra- and infraorbital nerves not previously described in medical literature.

## Figures and Tables

**Table 1:** Pathologic and immunophenotypic features useful in the differential diagnosis of Schwann cell neoplasms [15].

	Neurofibroma	Schwannoma	MPNST
*Cytology*			
Nuclear size	+	++	++/+++
Nuclear hyperchromasia	+	++	+++
Wavy nuclei	+++	+	++
*Histology*			
“Shredded carrot” type collagen	+++	−	−/+
Capsule	−	+++	−
Hyalinized vessels	−/+	+++	−
Fascicular growth pattern	−/+	++	+++
Mitotic activity	−/+	−/+	+++
Necrosis	−	−/+	+++
*Immunohistochemistry marker*			
S100 protein	++/+++	+++	+/++
Collagen IV	++/+++	+++	+/++
EMA	+	−(capsular)	−
CD34	+++	+++	++
Neurofilament protein	++	+(capsular rare intratumoral axons)	+/+++
Podoplanin	+	++	+
Calretinin	+	+++	NA
Sox10	+++	+++	+/++

MPNST: malignant peripheral nerve sheath tumor; EMA: epithelial membrane antigen; NA: not applicable.

## Data Availability

The data that support the findings of this study are available from the corresponding author upon reasonable request.
